# Supplementary material for: Are carbon emissions trading and green financial instruments synergistic? -Comprehensive quantitative research based on content analysis
Source: PLoS One. 2024 Mar 7;19(3):e0298601. doi: 10.1371/journal.pone.0298601 (PMC10919601; doi:10.1371/journal.pone.0298601)
Supplement: S1 Data — (ZIP) [file pone.0298601.s001.zip › Data/地级市Z_F工作报告.zip!/╕ú╜¿/╒─╓▌╩╨/└┤╫╘╣1⁄2╓┌║┼í╛┬φ┐╦╩2╛▌═°í┐_2013.docx]

各位代表：

　　现在，我代表漳州市人民政府向大会作政府工作报告，请予审议，并请市政协委员和列席人员提出意见。

　　一、2012年工作回顾

　　过去的一年，在省委、省政府和市委正确领导下，在市人大和市政协监督下，全市上下以科学发展观为指导，认真贯彻党的十七大、十八大精神，按照市第十次党代会部署，全力推进“工业化提速、城市化提升、现代农业提效”，经济社会发展取得新成效。

　　（一）综合实力再上台阶。地区生产总值完成2017.8亿元，增长12.6%。公共财政总收入205.48亿元，增长17.7%；其中地方公共财政收入131.71亿元，增长17.5%。全社会固定资产投资1486.9亿元，增长33.3%。农业总产值558.85亿元，增长4.5%。规模工业增加值750.21亿元，增长16.9%；规模工业总产值2680.53亿元，增长17%。外贸出口69.9亿美元，增长7.7%。实际利用外资8.9亿美元，增长0.3%。社会消费品零售总额621.06亿元，增长17.3%。城镇居民人均可支配收入23951元，增长13.3%；农民人均纯收入10389元，增长13.8%。多数指标增幅位居全省前三位。

　　（二）产业结构持续优化。工业支撑作用进一步增强。实施工业“千百工程”项目1068个，完成工业投资695.6亿元，增长30.4%。新增规模工业企业172家，占全省新增数近1/3。新认定高新技术企业27家，高新技术产业产值700亿元，增长14%。南靖高新区获批更名为漳州高新区。漳州钟表出口基地成为国家级外贸转型升级示范基地，获评“中国钟表之城”。新增中国驰名商标10件，增长150%。现代农业进一步增效。经检验检疫农产品出口货值41亿美元，增长23.2%；其中水产品出口26.5亿美元，增长39.5%。荣获“世界食用菌罐头之都”称号。新增产值10亿元以上农业产业化龙头企业3家、绿标产品5个、有机产品5个、无公害农产品28个。水利投资20.1亿元，增长108%，枋洋水利枢纽、古雷引水工程等重大水利基础设施项目开工建设。第三产业发展进一步提速。文化产业增加值80亿元，增长31%。摄制《市长带你游》，积极参加全省旅游促销活动，引进北京首旅集团开发建设南靖土楼景区，灵通山获评国家级风景名胜区，“桥上书屋”入选全球八大环保建筑，旅游总收入150亿元，增长22%。建筑业实现产值247.9亿元，增长45.3%。房地产完成投资254.5亿元，增长14%。金融业加快发展，新增分行3家、支行17家，全年本外币贷款余额1212.63亿元，增长20.3%；存款余额1525.74亿元，增长19.4%。举办武汉、西安“漳州名优产品展洽会”，参加新加坡亚洲国际食品展，产品市场进一步拓展。三次产业结构由16.6∶47.3∶36.1优化为15.9：47.9：36.2。

　　（三）“五大战役”成果丰硕。“五大战役”分别完成投资860亿元、682亿元、287.9亿元、466.5亿元、323.8亿元，均提前一季度完成年度目标。79个省级在建重点项目投资504.9亿元，完成计划200.1%。215个“十大竞赛”项目投资554亿元，完成计划205.4%。重大生产性项目建设进展顺利，4个投资超百亿元项目建成或基本建成。古雷石化启动项目、福欣特钢即将试投产，联盛纸业2条生产线投产，旗滨玻璃第5、6条生产线点火投产，第7、8条生产线开工建设。14个投资10亿元以上生产性项目投产或部分投产。重大基础设施建设进展顺利，在建高速公路里程348公里，建成国省干线98公里、农村水泥路400公里，改造危病桥40座。厦漳同城大道、沿海大通道、漳永高速、新江东大桥等项目开工建设，龙厦高铁、厦漳高速公路扩建工程、九龙江大桥、省道东东线建成通车，古雷30万吨级码头、漳州开发区8-10#万吨级码头基本建成，全年完成投资121亿元，增长36.6%。电力建设完成投资15.8亿元，增长28.5%。重大项目前期工作进展顺利，古雷南2#码头项目获得核准，漳州石化基地纳入国家《炼油产业发展规划（2011-2015）》，古雷炼化一体化、云霄核电、厦漳城市轻轨、中海油LNG、厦漳海底隧道等一批重大项目前期工作取得实质性进展。融资难问题得到有效缓解，海魁水产、万晖洁具成功上市；获批发行中期票据、企业债券等债务性融资102.3亿元；15个项目BT融资约50亿元。入选国家知识产权质押融资试点城市。

　　（四）“三维”招商又开新篇。举办世界500强和跨国公司“投资漳州”（北京）推介会、创业漳州台商大会、深圳民企项目洽谈会等招商活动，开展“世界500强企业漳州行”，参加中美企业投资合作论坛、中国美国商会年会，向世界500强、跨国公司和行业龙头企业推介漳州，促成荷兰孚宝等一批跨国企业到漳投资。全市列入省级跟踪对接“三维”项目238个，总投资4513亿元，其中投资10亿元以上项目81个、百亿元以上项目15个。积极探索专业化、市场化、特色化办会新路子，注重“展洽结合、展销结合、展游结合”，“农博会•花博会”共签订购销订单27.6亿元，签约合同项目103个，总投资328.1 亿元，奠基开工剪彩项目632个、总投资1035亿元。联想科技城、海投科技创业园、蓝恒达离子膜烧碱、联东U谷等一批投资超百亿元项目当年签约当年开工。

　　（五）城乡面貌明显改观。制定出台《关于进一步加强城乡规划工作的若干意见》，邀请国内外一流专家参与修编27个专项规划，完成347个村庄规划编制任务，实现村镇两级规划全覆盖。首次土地例行督察验收顺利通过，并被确定为“联创齐争”试点市。全年获批建设用地4.57万亩。启动城市建设“五年行动计划”，实施“十大工程”，投资60亿元建设城市基础设施和改造提升城市交通出入口，新改扩建城市道路173公里。开工建设奥林匹克体育中心等省运会重要比赛场馆。市区内河整治取得阶段性成效。拆除市区1000块约2万平方米户外广告牌，实现胜利路、迎宾路无跨街和高立柱广告。规划建设“一环、两带、七主题”总面积100平方公里的郊野公园22处，建成西溪湿地公园、林语堂文化园蕉园观光栈道和102.5公里绿道，新增绿地4527亩。完成百花村店面改造和梧浦村-迎宾路-百里花卉走廊-东南花都沿线景观提升、道路改造，城市形象明显提升。深入开展新农村示范村建设，以沈海高速公路沿线18个村和农村社区规划建设示范点为重点，强化山边、路边、宅边、水边的绿化和整治，涌现出长泰上蔡村、漳浦溪坂村等先进典型。“田园都市、生态之城”建设初见成效并受到广泛赞誉。国家卫生城市通过命名复审。城市文明指数测评从全国第51位上升到第8位。

　　《厦漳泉大都市区同城化发展总体规划纲要》编制完成，第一批18个同城化启动项目已完成10个，召开厦漳泉第二次党政联席会议，筛选确定第二批同城化项目39个。

　　（六）县域经济活力呈现。龙海入选全省县域经济实力“十强”和全国县域经济“百强”。漳浦、东山、长泰、华安入选全省县域经济发展“十佳”。漳浦荣获“全国粮食生产先进县”称号，平和被授予“中国地理标志商标第一县”称号，云霄获评“中国温泉之乡”。长泰、南靖通过国家生态县考核验收。诏安金都海洋生物产业园被确定为国家科技兴海产业示范基地。国家级漳州台商投资区获批设立。华侨农场体制机制进一步理顺。

　　（七）投资环境日臻完善。全面完成市县两级政府机构改革任务；开展新一轮行政审批项目清理，实现行政审批“三个百分百”；市级行政审批事项一次性压减211项，成为全省审批项目最少的设区市；加快政务服务体系标准化建设，市行政服务中心获批成为“全国服务业标准化试点单位”；推出43条项目审批提速增效的具体措施，建立“马上就办”工作制度，“五大战役”项目审批时限平均缩短146个工作日。

注重发挥政策效应，争取省政府出台《关于支持漳州石化基地加快开发建设十二条措施》，制定出台支持科技创新、海洋经济、旅游、中小企业、建筑业、民营经济、总部经济等发展的18项政策措施，采取“一企一策”，开展“两帮两促”活动，为企业排忧解难，取得明显成效。

　　（八）社会事业协调发展。全市财政民生支出164.4亿元，增长20.2%。6类55项为民办实事项目基本完成年度目标。城镇新增就业5.3万人，新增转移农村劳动力就业7.18万人，城镇登记失业率2%。社保扩面任务提前完成，发放社保卡420万张，城乡居民养老保险制度实现全覆盖。城乡居民基本医疗保险补助标准、大病医疗费用统筹基金支付比例进一步提高。出台《关于全面推进医疗卫生资源建设的若干意见》，新增床位2570张，漳州市第三医院投入使用，建成468所村卫生所。县级公立医院改革试点稳步推进，新农合参合率达99.8%。深入实施“生育文明•幸福家庭”促进计划，人口计生综合水平位居全省第二，人口自然增长率8.07‰。

　　教育惠民政策全面落实，建成校安工程项目448个，新改扩建50所公办幼儿园，新增11所达标高中校，225所义务教育标准化学校通过验收，更换国标校车216辆，云霄光电学院开工建设。完成国有文艺院团和广电网络体制改革，“海上丝绸•漳州史迹”项目列入世界文化遗产预备名录，漳州木偶列入世界非物质文化遗产优秀名录，电视连续剧《大航海》开机，央视《寻宝》、《乡村大世界》走进漳州。建成一批文化馆、图书馆、博物馆、数字电影院、乡镇文化综合站，实现全市行政村农家书屋全覆盖。市档案馆晋升为国家二级综合档案馆。体育事业取得新成绩，28个省运会场馆加快建设，建成1667个农民健身工程，实现全市行政村健身工程全覆盖。

　　开工建设各类保障性住房20989套，基本建成17438套，超额完成省定目标。制定保障性住房建设管理相关制度，确保配租配售公开、公正，经验做法在全省推广。投资6.55亿元完成新一轮农网升级改造。建成农村公路安保工程640公里。国家级“生态市”和“森林城市”创建活动深入开展，建立生态公益林储备库试点，节能减排指标控制在省下达任务范围内。九龙江流域（漳州段）Ⅲ类水质达标率96.9%，县级以上集中式饮用水源水质达标率100%。扶贫开发力度进一步加大，投入3.4亿元完成2081户、8000人“造福工程”年度搬迁任务。出台《关于进一步支持和促进革命老区加快发展的若干意见》，市财政安排3000万元支持老区发展特色产业。老龄工作扎实推进，建成居家养老服务站242个，实现全市社区全覆盖。

　　坚持领导干部定期接访下访制度，成立市级联合接访调解中心。推广华安县新圩村“一圩一日”集中办事和诏安县倾听群众诉求常态化做法，把问题化解在萌芽状态。深入开展道路交通安全和安全生产专项整治行动，交通事故和安全生产控制指标全面下降。切实加强社会管理综合治理，全面完成综合警务改革，深入开展严打整治专项行动，深化社区矫正、安置帮教和法律援助工作，社会保持安定稳定，全市社会治安满意率达97.1%。国防动员工作取得新成效，实现创建全国双拥模范城“五连冠”。开展新一批荣誉市民评选授荣。治理“餐桌污染”、建设食品放心工程成效显著。圆满完成村（居）委会换届选举。妇女儿童、民族宗教、外事侨务、新闻出版、社会科学、行政复议、地方志、档案、地震、气象、残疾人、红十字会、统计、人防、物价等各项工作都取得新的进步。

　　（九）政府运行更加规范。认真执行人大及其常委会决定决议，自觉接受人大及其常委会和政协的监督，积极配合人大、政协开展检查、视察和调研活动，广泛听取市人大、市政协和各民主党派、工商联、各人民团体和社会各界人士意见建议，全年共办理市人大代表建议232件、市政协提案280件，办结率均为100%。深入开展“作风建设年”和“万名机关干部结对帮扶困难群众”活动，完善政府绩效评估，加大“庸懒散”治理力度。电子政务建设迈出新步伐，政府门户网站全新改版，市长信箱启动运行。严格执行党风廉政建设责任制，强化行政监察、审计监督，坚决纠正损害群众利益的不正之风，政风行风进一步好转。开展海域资源市场化配置工作，经验在全省推广。

　　各位代表，回顾过去的一年，成绩令人鼓舞。这是省委、省政府和市委正确领导的结果，是全市人民团结一心、奋力拼搏和各方面共同努力的结果。在此，我谨代表市人民政府向为漳州发展付出辛勤劳动的全市人民，向给予政府工作大力支持、有效监督的人大代表、政协委员、各民主党派、工商联、各人民团体、无党派人士、离退休老同志和社会各界人士，向积极服务漳州发展的中央、省驻漳机构、驻漳部队、武警官兵、公安民警，向关心支持漳州发展的港澳台同胞、海外侨胞和国际友人，表示崇高的敬意和诚挚的感谢！

　　在总结成绩的同时，我们也清醒地看到，当前漳州经济社会发展仍面临不少困难和问题，主要是：经济总量偏小，产业结构不尽合理，自主创新能力不强；财政收支矛盾依然突出；中心城市功能配套不够完善，辐射带动能力不足；农业农村基础设施较为脆弱；受外部环境影响，经济下行压力加大，外经贸形势不容乐观；资源环境对经济发展的制约进一步加大；交通安全和安全生产等方面工作有待进一步改进；一些公务人员的思想观念、知识结构、工作作风还不适应科学发展观的要求，消极腐败现象和不正之风在一些领域仍然存在。我们要高度重视这些问题并认真加以解决。

　　二、扎实做好2013年的工作

　　党的十八大对全面建成小康社会作出了重要部署，对政府工作提出了新的更高要求。根据市委统一部署，2013年政府工作的总体要求是：深入学习和全面贯彻党的十八大精神，坚持以邓小平理论、“三个代表”重要思想、科学发展观为指导，全面落实中央经济工作会议、省委九届六次全会和市第十次党代会的部署，紧紧围绕主题主线，以提高经济增长质量和效益为中心，坚持稳中求进、好中求快，突出“工业化提速、城镇化提升、现代农业提效”，全力打好“五大战役”，深入开展“十大竞赛”，着力产业发展，着力城乡建设，着力改善和保障民生，着力生态文明建设，着力改革开放，着力转变作风，实现经济持续健康发展和社会和谐稳定。

　　经济社会发展的主要预期目标为：地区生产总值增长12%左右；公共财政总收入增长15%，其中地方公共财政收入增长15%；规模工业增加值增长16%，规模工业总产值增长18.9%；农业总产值增长4.1%；全社会固定资产投资增长22%；外贸出口增长5%；实际利用外资增长5%；社会消费品零售总额增长15%;居民消费价格总水平涨幅控制在3.5%左右；城镇登记失业率控制在4%以内；人口自然增长率控制在8.1‰以内；城镇居民人均可支配收入增长13%，农村居民人均可支配收入增长14%;节能减排指标控制在省政府下达范围之内。

　　围绕上述要求和目标，必须注重抓好九个方面工作：

　　（一）注重“两轮”驱动扩内需

　　扩大有效投资。强化抓项目就是抓产业、抓项目就是抓发展的理念，深化“五大战役”内涵，拓展“十大竞赛”领域，进一步壮大实体经济。更加突出工业和基础设施项目建设，继续推进工业“千百工程”，力争全年建设投资2000万元以上工业项目1000个，投资亿元以上工业项目300个，投产项目400个，工业投资突破700亿元；加快“一桥两港两铁三道五路”建设，力争完成投资130亿元以上，初步形成“两纵两横五联”高速公路网和“三纵三横五联”国省干线公路网。加大电力基础设施投入，力争完成投资20亿元以上。

　　强化“三维”对接。密切跟踪央企、外企、民企投资动态，增强招商引资实效性，提高“三维”对接落地率。用好省政府《关于加快引进世界500强企业十条措施》，以龙头企业落地促进链条延伸、产业集群。加强与海外华人财团的联系，提升漳港澳合作层次和水平，深入实施“回归工程”。兑现扶持政策，鼓励上市企业返程投资。坚持“专业化、市场化、特色化”方向，办好“农博会•花博会”、漳台经贸恳谈会，积极参加第17届中国投洽会，进一步提高办会水平和招商实效。

　　促进居民消费。开展“市场建设年”活动，加快城市综合体、商贸广场、农贸市场建设，培育一批餐饮酒店、时尚消费、休闲购物、娱乐健身等商业街区。落实调整收入分配的政策措施，坚持“两同步、两提高”，努力增加城乡居民收入。进一步整顿和规范市场经济秩序，深入开展食品、药品安全整治，严厉打击制假售假、商业欺诈、盗版侵权等违法行为，切实维护消费者合法权益。

　　（二）注重园区建设和创新驱动

　　更加突出园区在工业化提速中的主力军作用，向园区要速度要效益要质量。提升规划水平，高标准编制空间和产业规划，科学构筑发展框架；加强政策扶持，研究制定差异化政策，在资金投入、土地储备、项目引办、权限下放等方面给予更大支持；创新体制机制，强化“小政府、大社会”、“小机构、大服务”导向，构筑精简、统一、效能的政务环境，着力增强吸纳要素、承载项目、集聚产业的能力。高标准规划建设漳州（古雷-南太武）滨海新区。全力推进古雷开发建设，用好用足先行先试各类优惠政策，确保石化启动项目顺利投产，推动炼化一体化和一批中下游项目尽快落地投建，进一步完善园区整体配套，加快打造“世界一流现代化临港石化产业基地”。高品位建设台商投资区，积极承接台湾产业整体转移，扎实推进福欣特钢、联盛纸业投产达产，加快海峡商务营运中心等项目建设，高标准规划，全面完善市政基础设施配套，努力建成环境优美、宜居宜业的漳州新城区。漳州开发区要在新的起点上加强产城互动、港城联动，大力发展以临港工业、临港物流业和滨海旅游业为主的海洋经济，努力做大做强经济总量。支持漳州高新区积极申报国家级高新技术产业园区,努力打造高新技术企业汇集、高科技人才集聚、产业特色鲜明的高新技术产业密集带。常山开发区要突出侨的特色，全力打造海西滨海华侨城。支持南溪湾经济开发区等申报省级经济开发区，创造条件推动更多开发区做大做强。力争全市各类园区工业产值、税收收入均增长20%以上。

　　实施创新驱动发展战略，增强科技创新在提高社会生产力和综合实力中的战略支撑。深入贯彻《关于深化科技体制改革 加快创新体系建设的实施意见》,力争全市高新技术企业突破100家，高新技术产业产值突破800亿元。强化企业主体地位，争取新设立6家以上省级企业工程技术研究中心。开展“工业技改年”活动，出台技改专项扶持政策，实施“百项百亿”技改工程，完成技改投入250亿元以上。推进“一城二谷三园四中心”建设，增强产业支撑。加快“数字漳州”建设，推动信息化和工业化互动融合。加大“蓝火计划”推进力度，实施产学研“双十双百”行动，充分用好“6•18”项目成果交易平台，引进产学研科技成果400项以上。加快实施“三个百名”人才自主培养计划，积极引进高端人才，加速壮大创新人才队伍。

　　（三）注重培育新的经济增长点

　　发展壮大海洋经济。全面落实《关于加快海洋经济发展的实施意见》，进一步完善细化扶持政策。围绕石化、钢铁、船舶、汽车、装备制造、光伏玻璃等产业，加快引办一批高端化、高质化、高新化临港工业项目，加快形成一批特色鲜明、配套完善的产业集群。以国家海洋三所漳州科技兴海基地、诏安金都海洋生物产业园、东山海洋生物科技产业园等为平台，积极发展海洋生物医药、海洋服务、海水淡化与综合利用、水产品精深加工等海洋产业，培育海洋经济新业态，发展壮大特色产业群体。支持发展远洋捕捞和工厂化海水养殖，提升现代海洋渔业发展水平。加快推进港口码头和渔港建设，增强港口承载能力。

　　加快发展现代物流产业。抓好国务院降低流通费用10项政策措施的落实。积极推进荷兰孚宝、青岛啤酒物流仓储、盛辉公路港等项目落地开工，着力发展以第三方物流为标志的产业物流。规划建设一批现代物流园区、物流配送中心，引进和培育物流龙头企业。扶持闽南商业综合批发市场、汽车交易市场、家具建材市场三大市场集群发展，加快海峡两岸（国际）农产品物流城、海峡两岸花卉集散中心、海峡两岸（东山）水产品加工集散基地、漳州花卉交易中心、芗城闽南汽车交易城、闽粤（平和）农资物流交易大市场、南靖兰花批发市场建设，启动建设农资物流园，规划建设食品、钢铁等专业市场，逐步搭建“买世界、卖世界”的大营销格局。加大电子商务政策扶持力度，筹划建设电子商务产业基地，推动网络市场和实体市场共同发展。

　　努力提升旅游、文化产业。深入落实《关于加快旅游产业发展的实施意见》，加快推进旅游综合体和高星级酒店建设，增强旅游服务接待能力；积极引进旅游大企业大集团，推进东南花都、南靖土楼、白塘湾等一批重大旅游项目建设，支持有条件的景区创建4A 、5A级旅游景区，形成龙头带动效应；大力开发乡村旅游产品，重点打造10个以上乡村游示范点；加强创意旅游产品开发，做大旅游购物市场；以厦深动车开通为契机，深化区域旅游协作，加强旅游营销，推介精品线路，拓展旅游客源市场。加强文化市场建设，积极推进一批文化产业园区、文化产业项目和工业设计、产品包装项目建设，培育一批骨干企业，增强文化产业实力和竞争力。

　　（四）注重促进农业增效、农民增收

　　增强农业综合生产能力。扶持发展八大优势产业，鼓励发展林下经济，加快形成集中连片、优势突出、特色明显的产业带。开展农业产业化龙头企业“十佳”、“十优”评选，促进企业发展壮大。推进农产品精深加工，深化“五新”推广，扶持种业发展，建设10个食品加工示范区、10个农业“五新”推广示范基地和10个省级新品种核心示范片。提升漳浦台湾农民创业园，建设省级农民创业园，支持各县（市）创办农民创业园。大力发展“三品一标”，创建农产品质量安全监管示范县（区）、示范乡镇，健全完善农产品质量安全可追溯体系，筹建区域性农产品检测中心，构建从田间到餐桌的食品安全链条。

　　改善农业基础设施。加强以水利为重点的农村基础设施建设，加快九龙江防洪工程、枋洋水利枢纽、古雷引水工程、市区第二水源、东山岛二水源等重点项目建设，开工建设7个重大水利项目和271个农村水利项目，抓好3个小型农田水利重点县建设，全年投资力争超过20亿元。实施设施农业提升工程，新增设施农业2.85万亩，建设高标准基本农田5.8万亩。加强水土流失治理，深入推进5个省级重点县和一批重点镇水土流失治理工作。抓好菜篮子工程，加大城市副食品基地建设力度。推进新一代气象雷达项目建设，实施人工影响天气保障工程。

　　深化农村综合改革。落实强农惠农富农政策。积极创新农业生产经营体制，充分保障农民土地承包经营权，健全土地流转服务体系，发展多种形式规模经营。扶持发展农民专业合作社，培育专业大户和经纪人队伍，着力发展多种形式的新型农民合作组织和多元服务主体，努力构建集约化、专业化、组织化、社会化相结合的新型农业经营体系。推进乡镇“三农”服务中心规范化建设，发挥驻村干部、农村“六大员”等骨干服务力量作用，完善基层公共服务体系。实施结对帮扶，扶持壮大村级集体经济。推进政策性农业保险，增强农业抗风险能力。

　　（五）注重提升城市化、城镇化水平

　　强化规划引领。编制规划导则，构建“田园都市，生态之城”支撑体系。坚持“四规合一”，严格按照标准进行规划设计、开发建设。加快漳州城市总体规划（2012-2030年）成果编制和报批，抓紧修编和完善城市景观、公共服务设施、道路交通、漳州水城、森林城市、夜景工程、城区防涝等专项规划，修编395个村庄规划，抓好控规单元规划编制，实现中心城区和省、市试点镇控规全覆盖。组织实施《厦漳泉大都市区同城化总体发展规划》，抓紧编制各专项规划。

　　完善城市功能。继续实施城市建设“五年行动计划”和“十大工程”，加快推进碧湖生态园、圆山新城等重点区域开发建设。深入开展内河综合整治和城乡环境“点线面”综合整治，实施城市彩化亮化工程，提升拓展郊野公园建设成果，建设荔枝海公园、水仙花公园和一批城市片林，持续提升城市绿地率。充分挖掘地方文化特质，彰显现代文化气息，加快“三馆一中心”、漳州古城文化旅游综合体、文化创意园规划建设，举办“漳州国际公共艺术展”，打造城市文化平台。新建一批道路，打通“断头路”，改造优化交叉路口，完善城市路网。加强道路交通组织，优先发展城市公交，新增150辆新能源公共汽车，新建一批立体停车楼、停车场，开辟一批市民活动广场，着力解决市民行车难、停车难和活动空间小等问题。加快打通中心城区与县域之间快速通道，推进漳华路、圣王大道等道路建设，畅通人流物流。鼓励发展总部经济、楼宇经济等新兴业态，增强产业支撑。

　　加快县域发展。研究出台差别化、具体化扶持政策，拓展县域发展空间。统筹县域产业发展规划，努力形成“一县一业”、“一镇一品”发展格局。引导县城拓展新区、改造老城、完善功能、彰显特色。把发展民营经济作为富民强县的重要抓手，健全扶持民营经济和小微企业发展的服务体系，落实提高小微企业增值税和营业税起征点等各项优惠政策，出台知识产权质押融资扶持政策，扩大中小企业质押融资规模；引导企业靠大联强，鼓励开展兼并重组，培育壮大一批民营大企业大集团。

　　创新体制机制。支持东山、长泰开展城乡一体化试点工作，深化小城镇综合改革，统筹安排户籍、土地、财税、住房、教育、医疗、社会保障等体制、政策、利益调整，有序推进农业转移人口市民化。推动基本公共服务从户籍人口向常住人口覆盖，从城市社区向小城镇和农村延伸，促进公共资源均衡化配置。深化创建全国文明城市活动，创新城市管理体制，加快数字化城管项目建设，开展“六项专项整治”，依法拆除违法建筑，规范提升广告管理，组织“六个一批”评选活动，打造规范整洁、文明有序的城市形象。深入研究同城政策，积极争取厦门综合配套改革试验区优惠政策向漳州延伸。

　　（六）注重深化改革扩大开放

　　深化漳台合作。全面落实《关于进一步鼓励台商创业和支持台资企业发展的若干意见》，维护台商合法权益，支持在漳台商发展壮大，加强两地产业园区对接合作，推动台湾石化产业、高新技术、现代农业、新兴产业等产业组团式转移。挖掘祖地文化、宗亲文化内涵，加快建设一批对台文化交流合作基地，拓展文化旅游、文化会展、动漫游戏等方面的合作，共同打造特色文化产业。积极承办海峡论坛活动项目，扩大青少年、文化教育、民间信仰、特色乡镇、族谱对接等基层、民间交流。

　　扩大外贸出口。继续实施鼓励扩大出口的扶持政策，完善重点外贸出口企业联系和对口帮扶机制，加快出口退税速度，进一步优化通关环境，努力扩大外贸出口。支持企业参加出口企业信用保险，扎实推进国家科技兴贸创新基地和省级出口基地建设，积极建设质量安全示范区，提升产品竞争力。加快一批出口型生产性项目投建投产速度，注重品牌质量和效益，促进企业转型升级，培育新的外贸增长点。支持企业参加境内外大型展会，努力拓宽营销渠道，拓展外贸市场。

　　创新资本运作。支持九龙江、福建漳龙、城投、交通运输集团等国有融资平台做大做强。积极运用BT、BOT、融资租赁等方式推进重大基础设施、公共设施项目建设；鼓励发行集合债、建设债券、中短期票据等，拓宽投融资渠道；积极争取信贷支持，确保新增贷款200亿元以上。支持发展地方金融机构，争取更多海内外金融机构入驻。加大企业上市扶持力度，力争新增上市企业6家。完善政府资金统筹机制，继续扩大政府预决算公开范围和内容，提高预算绩效管理水平。

　　推进各项改革。加快事业单位分类改革。进一步深化行政审批制度改革，推进行政审批项目和公共服务项目标准化建设，不断提高行政效能和服务效率。深化国有企业改革，积极推动市属国有或国有控股企业高位嫁接央企、大型国企。继续做好“营改增”试点工作。扩大公立医院综合改革试点范围，引进社会资本兴办各类医疗机构，加快形成多元化办医格局。进一步推进科技、教育、文化等领域改革。认真组织实施第三次全国经济普查。

　　（七）注重生态文明，建设美丽漳州

　　加强生态环境保护。加快国家级生态市创建步伐，确保2013年底全市50%的县(市、区)达标。深入开展国家森林城市创建活动，扎实推进“六大森林工程”、“四绿”工程和城市生态绿化等建设，完成造林绿化40万亩以上。继续实施“碧海银滩”工程，推进海洋环境保护与生态修复。加强自然保护区、无人海岛、重要湿地、沿海防护林、“三沿一环”等重点生态功能区的强制性保护，构建生态安全屏障。完善生态补偿机制，落实最严格的耕地保护制度、水资源管理制度和环境保护制度。加强生态文明宣传教育，增强全民节约意识、环保意识、生态意识。

　　推进美丽乡村建设。以“生产发展、生活富裕、生态良好”为目标，以新农村示范村和村容整治示范村为重点，把村庄当作景区、景点来规划设计，努力建设和谐幸福的美丽乡村。从改善人居环境入手，引导人畜分离、雨污分流、垃圾分拣、生产与生活分开，深入推进农村家园清洁行动，深化农村环境连片整治。实施农村危房改造，整治提升蘑菇房。实施新一轮扶贫开发和“造福工程”，落实3个省级重点县、37个重点村扶贫开发任务，扶持财政困难县、库区移民后发地区、老少边贫地区和民族乡村加快发展。

　　提高综合整治水平。以解决损害群众健康的突出环境问题为重点，强化各类污染防治。扎实做好重点流域水环境综合治理，健全河道采砂长效治理机制，确保九龙江流域（漳州段）Ⅲ类水质和市县两级集中式饮用水源水质稳定达标。制定PM2.5监测方案，开展大气污染防治工作。突出抓好重金属污染防治，加强危险废物和医疗废物处置全过程监管。持续推进餐饮油烟、生活噪声、建筑工地粉尘等专项治理。

　　完成节能减排目标。认真落实建设项目节能评估审查和环境影响评价、环保“三同时”等制度。抓好十大重点领域节能重点工程项目建设，按期完成行业落后产能淘汰任务。加强重点行业脱硫脱硝设施监管，确保达标排放、有效减排。积极推广LED节能灯等绿色产品，培育壮大节能环保产业。狠抓工业点源、农业面源和生活污染源治理，提高工业废水和重点乡镇污水、垃圾集中收集处理能力。

　　（八）注重民生改善，让人民群众过上更好生活

　　健全社会保障体系。积极拓宽就业渠道，确保新增城镇就业5万人以上，转移农村富余劳动力6万人以上。推动社保、医保、低保提标扩面，提高城乡居民医疗保险财政补助标准，开展城乡居民大病保险，力争住院政策补偿比达到75%，住院最高封顶线不低于10万元。完善失地农民社会养老保险，提高企业退休人员基本养老金，推动城乡居民社会养老保险制度一体化。市财政安排1400万元补贴全市村主干。加快推进金保工程建设，提高信息化水平。加快保障性安居工程建设，新开工建设10859套，争取建成或基本建成7400套。抓好乡镇养老院、农村“幸福园”、社区居家养老服务中心等养老机构建设。保障妇女、儿童和劳动者合法权益，大力发展社会福利、社会救助、社会优抚、社会慈善、红十字和残疾人事业。

　　加快发展各项社会事业。按照“统筹规划、分步实施、重点突破”的原则，有计划地解决社会事业发展长期积累的深层次矛盾。实施医疗卫生事业“三年行动计划”，努力解决群众看病难、看病贵、看病烦问题。开展“医疗卫生事业建设年”活动，推进十大重点卫生医疗项目建设，新增床位2000张、卫技人员1000名以上，培训全科医生和乡村医生4000名以上。加强医疗服务管理，提升优质服务水平。实行社保卡就诊“一卡通”，做到住院费用即时结报。继续推进药品零差率销售，努力实现全覆盖。坚持计生基本国策，深入开展“生育文明•幸福家庭”创建活动，努力提高人口素质。

　　推进教育资源向农村地区、贫困地区、城市新区倾斜，促进教育均衡发展。新开工建设80个校安工程和18个城区中小学扩容工程，新改扩建42所公办幼儿园，更换国标校车57部。加快市实验小学古雷开发区分校建设，筹办漳州一中初中部，创建10所省级示范性特色高中学校。落实高校办学自主权，推动高校与企业合作办学。持续加强校园及周边治安综合治理。做大做强职业技术教育，扶持特殊教育，重视老年教育，发展学前教育、继续教育、社区教育，构建终身教育体系。

　　加快发展文化事业，完善文化服务体系，加强市县公共文化基础设施建设，培养壮大文化队伍，繁荣文学艺术创作。积极推动“海上丝绸之路”申遗工作，加强闽南文化生态、非物质文化遗产、民俗民间文化和历史文化名城、名镇、名村的保护开发。认真筹备第十五届省运会，加快体育场馆和接待酒店建设，加强体育竞训人员培训，确保按时保质完成建设和训练任务。精心办好第十二届市运会，大力开展全民健身运动，促进群众体育和竞技体育同步发展。继续抓好第二轮修志工作，支持市档案馆创建国家一级档案馆。

　　（九）注重加强和创新社会管理

　　加强基层社会管理和服务体系建设，全面推行城市社区网格化管理，增强社区服务功能。发挥维稳“三支队伍”作用，完善人民调解、行政调解、司法调解联动工作体系，妥善化解社会矛盾。健全维护群众权益机制，完善信访工作制度，畅通“12345”政务服务热线，引导群众依法合理表达诉求。创新流动人口、特殊人群、“两新”组织、网络虚拟社会的服务管理,实行居住证制度，推进社区矫正工作制度化、安置帮教工作规范化，建立健全重大决策社会稳定风险评估机制，深化平安建设，完善立体化社会治安防控体系，依法防范和惩治违法犯罪活动。做好“六五”普法和行政复议工作。

　　巩固提升企业安全生产标准化建设，深入开展道路交通安全综合整治“三年行动”，坚决遏制重特大安全生产事故发生。加强公共安全预防预警和应急处置体系建设。加强国防教育动员、民兵预备役、海防、打私、人防工作，深化“双拥”共建活动。做好新形势下的外事侨务、民族宗教工作。

　　三、建设人民满意的服务型政府

　　致力建设职能科学、结构优化、廉洁高效、人民满意的服务型政府，努力为全市人民安居乐业和广大投资者创业致富提供优质服务。

　　以执政为民的追求让人民更满意。始终把人民放在心中最高位置，坚持为大多数人民谋利益，坚持从群众最关心、最迫切的问题抓起，重点实施26项为民办实事项目，努力让人民有更好的教育、更稳定的工作、更满意的收入、更可靠的社会保障、更高水平的医疗卫生服务、更舒适的居住条件、更方便的出行、更优美的环境。

　　以高效务实的作风让人民更满意。贯彻落实中央关于改进工作作风、密切联系群众的八项规定。精简会议活动，改进文风会风，开短会、讲短话、发短文。全面推行“马上就办”，让企业、基层和群众少跑腿、少受累。深化机关效能建设，持续治庸治懒治散治奢，确保在岗在位在状态。完善绩效评估，严肃行政问责。

　　以规范透明的制度让人民更满意。加强和完善制度建设，用制度管权、靠制度管人、按制度办事。规范行政裁量权并加强后续监管，做到严格规范、公正文明。自觉接受人大依法监督、政协民主监督和社会舆论监督，强化政府层级监督和监察、审计等专门监督。深化电子政务和信息公开，创新网络问政平台管理模式、运用模式和建设模式，让权力在阳光下运行。

　　以清正廉洁的形象让人民更满意。执行国库集中支付制度，推行公务卡制度，严格控制“三公”经费支出，把有限的资源和财力用于改善民生、发展经济。全面落实党风廉政建设责任制，更加重视预防腐败，推动监管制度嵌入管理流程，做到源头介入、过程监督，切实防止权力寻租，努力实现干部清正、政府清廉、政治清明。

　　各位代表，党的十八大指引我们踏上新的历史征程，全面建成小康社会的宏伟目标催人奋进。让我们紧密团结在以习近平同志为总书记的党中央周围，在省委、省政府和市委的领导下，团结和依靠全市人民，凝心聚力，开拓进取，为加快建设创业创新、又富又美的新漳州而不懈奋斗！
